# Problems in peer relationships and low engagement in romantic relationships in preterm born adolescents: effects of maternal warmth in early childhood

**DOI:** 10.1007/s00787-024-02399-6

**Published:** 2024-03-16

**Authors:** Ayten Bilgin, Dieter Wolke, Hayley Trower, Nicole Baumann, Katri Räikkönen, Kati Heinonen, Eero Kajantie, Daniel Schnitzlein, Sakari Lemola

**Affiliations:** 1 Department of Psychology, University of Essex, Colchester, CO4 3SQ UK; 2 Department of Psychology, University of Warwick, Coventry, UK; 3 Division of Health Sciences, Warwick Medical School, Mental Health and Wellbeing Unit, University of Warwick, Coventry, UK; 4 Department of Population Health Sciences, University of Leicester, Leicester, UK; 5 School of Psychological Sciences, Turner Institute for Brain and Mental Health, Monash University, Melbourne, Australia; 6 Department of Psychology & Logopedics, University of Helsinki, Helsinki, Finland; 7 Psychology/Welfare Sciences, Faculty of Social Sciences, Tampere University, Tampere, Finland; 8 Finnish Institute for Health and Welfare, Helsinki, Finland; 9 Clinical Medicine Research Unit, Medical Research Center Oulu, Oulu University Hospital and University of Oulu, Oulu, Finland; 10 Pediatric Research Center, Children’s Hospital, University of Helsinki and Helsinki University Hospital, Helsinki, Finland; 11 Department of Clinical and Molecular Medicine, Norwegian University of Science and Technology, Trondheim, Norway; 12 Leibniz University of Hannover, Hannover, Germany; 13 IZA Bonn, Bonn, Germany; 14 Department of Psychology, Bielefeld University, Bielefeld, Germany

**Keywords:** Preterm birth, Maternal warmth, Social relationships, Adolescence, Millennium Cohort Study

## Abstract

This study examined whether maternal warmth in early childhood moderates the association between preterm birth and problems in peer relationships and low engagement in romantic relationships in adolescence. We studied 9193 individuals from the Millennium Cohort Study in the United Kingdom, 99 (1.1%) of whom were born very preterm (VPT; < 32 weeks of gestation) and 629 (6.8%) moderate-to-late preterm (MLPT; 32–36 weeks gestation). Maternal warmth was reported by the mothers when their children were 3 years old. Peer relationship problems were reported by both the participants and their mothers at 14 and 17 years. Further, participants reported their engagement in romantic relationships at 14 and 17 years. All outcome variables were z-standardized, and the moderation effect was examined via hierarchical linear regressions. Compared to full-term birth, both MLPT and VPT birth were associated with lower engagement in romantic relationships at 17 years of age (b = .04, p = .02; b = .11, p = .02, respectively), and VPT birth was associated with increased peer relationship problems at 14 (b = .29, p = .01) and 17 years of age (b = .22, p = .046). Maternal warmth in early childhood was similarly associated with lower peer relationship problems in MLPT, VPT and full-term born adolescents. However, there was no influence of maternal warmth on engagement in romantic relationships at 17 years of age. There is no major modifying effect of maternal warmth in early childhood on the association between PT birth and peer relationship problems and low engagement in romantic relationships at 14 and 17 years of ages.

Adolescence, defined as the period from ages 10 to 19, marks an important period of social transitions [1, 2]. During adolescence, the time spent with peers increases in comparison to childhood with a greater emphasis on peer acceptance and conformity, and peer relationships become more intimate [3]. Further, interest in romantic relationships starts in early adolescence with short-lived relationships characterized by group dating, which progresses into a single-committed, sexual and exclusive relationship in late adolescence [4, 5]. Considering the increasing time spent with peers, the likelihood of experiencing difficulties in peer and romantic relationships also increases during this period [6]. Difficulties in establishing and maintaining peer and romantic relationships may have long-lasting influences on the mental health and well-being of adolescents [7–9].

There is increasing evidence that individuals born preterm (PT: birth before 37 weeks of gestation), particularly those who are born very preterm (VPT: birth before 32 weeks of gestational age), are at an increased risk of experiencing problems in peer relationships during childhood and adolescence [10–14]. In addition, PT born individuals are less likely to form romantic relationships than full-term (FT: birth between 37 and 41 weeks) borns in adulthood, with these associations being stronger for those with a lower gestational age [15, 16]. The difference in forming romantic relationships for individuals born PT might already be evident in adolescence, however this remains currently unknown. Experiencing these problems in adolescence could increase the risk for developing mental health problems and difficulties during the transition into adulthood in PT born individuals [17]. Therefore, it is important to understand which factors might differentiate PT born adolescents who experience difficulties in peer relationships and engagement in romantic relationships from those who do not.

The quality of early parenting could positively influence the association between PT birth and problems in peer and romantic relationships. Positive parenting such as maternal warmth which includes displaying affection, comfort and providing nurturance and support for the child, may buffer against the adverse effects of PT birth [18]. This has been reported by several studies. To illustrate, infants born PT showed more positive outcomes in social skills than FT born infants when they were raised in a positive environment [19]. It was further shown that the levels of internalizing problems of PT children were similar to FT born children at 11 years of age when they had high maternal sensitivity at 18 months of age [20]. In addition, the academic performance of VPT born children and adolescents reached similar levels to FT born children when they had high sensitive parenting [21, 22]. However, it is unknown if positive parenting such as maternal warmth in early childhood can protect PT born adolescents from having problems relating to peer relationships and romantic engagement. Thus, the aim of the current study was to investigate whether maternal warmth in early childhood can buffer the adverse impact of PT birth on adolescents’ problems in peer relationships and romantic engagement. Two gestational age groups of preterm born children (i.e., moderate to late preterm: MLPT, birth between 32 and 36 weeks of gestation, and very preterm: VPT) are studied in comparison to those born FT. It was hypothesised that PT born adolescents will have a larger decrease in peer relationship problems and a larger decrease in low engagement in romantic relationships when maternal warmth in early childhood increases, while FT born adolescents will be less influenced by the levels of maternal warmth.

## Methods

### Participants

The current study used data from the Millennium Cohort Study (MCS), which is a nationally representative longitudinal study of 18,818 infants born in the United Kingdom (UK) [23]. A random sample of all infants born in England and Wales between September 2000 and August 2001, and in Scotland and Northern Ireland between November 2000 and January 2022, was drawn from Child Benefit registers that cover virtually all children in the UK. The sample is geographically clustered with over-sampling of ethnic minority and disadvantaged areas. The first sweep of interviews with cohort members’ mothers took place when the infants were 9 months old and follow up interviews were conducted when the children were 3, 5, 7, 11, 14 and 17 years of age. In the current study, we focused on the assessments made at 9 months, 3, 14 and 17 years of age. The interviews included questions on a wide variety of topics, including health, education, social, family, and economic status of the cohort members’ households. Detailed information on the sampling and scope of MCS is available at: http://www.cls.ioe.ac.uk/. Ethical approval and written informed consent for all participants were obtained (London—Hampstead Research Ethics Committee, REC reference 14/LO/0868). Ethical approval for the present study was obtained from the Ethics Committee at the University of Warwick (reference 96/17–18). The sample in the current study included 99 VPT, 629 MLPT, and 8465 FT participants whose information was available at 17 years assessment.

### Measures

#### Preterm birth

Gestational age in full weeks plus days was extracted from medical records as a continuous variable, which was recoded into a categorical variable according to the following birth groups: very preterm (< 32 completed weeks of gestation); moderate to late preterm (32–36 completed weeks of gestation); full-term (37–41 completed weeks of gestation).

#### Maternal warmth

Mothers reported on their level of warmth using seven items of the Pianta child-parent relationship scale at 3 years of age [24]. The scale includes items such as the following: ‘*I share an affectionate, warm relationship with the child’*; ‘*Child will seek comfort from me*’ (Please see Supplementary Table 1 for a full detail of the measures). Each item was scored as follows: 0 = definitely does not apply, 1 = not really, 2 = neutral, 3 = applies sometimes, or 4 = definitely applies. Can’t say responses were considered as missing information. Scores were summed for mothers who had completed all items, which had a minimum of 0 and a maximum of 28. This is a widely used scale for measurement of maternal warmth in large-scale longitudinal studies with scores below 22 suggesting low maternal warmth [25]. The alpha coefficient for the maternal warmth score is 0.68.

#### Peer relationship problems

Participants reported on peer relationship problems with the following one items at 14 years: *“How often other children hurt or pick on you?”*. The response scale ranged from 1 = Never to 6 = Most days. At 17 years of age, participants reported on peer relationship problems using the 5-item peer relationships subscale of the Strengths and Difficulties Questionnaire (SDQ) [26]. The items (e.g., ‘*Picked on or bullied by other children”)* were rated on a three-point scale: 0 = Not true; 1 = Somewhat true; 2 = Certainly true. The SDQ is a widely used and reliable questionnaire for both research and clinical purposes [27]. In addition to the self-reports, mothers reported on the peer relationship problems of their adolescents at 14 and 17 years of age using the peer relationships subscale of the SDQ [26]. The mean of 5 SDQ items reflecting peer relationship problems were computed separately for self- and mother- reports.

#### Low engagement in romantic relationships

Participants reported on their engagement in romantic relationships at 14 years of age with the following four items: ‘*Do you have a boyfriend/girlfriend?’, ‘Have you held hands with another young person?’, ‘Have you kissed another person?’, ‘Have you cuddled with another person?’, and at 17 years of age with the following two items: ‘Have you ever had sexual intercourse?’, ‘Do you have a boyfriend/girlfriend?’*. The response scale was as follows: 1 = Yes; 2 = No. Four items at 14 years, and two items at 17 years were summed up to create a continuous variable at each assessment point with higher scores reflecting low engagement in romantic relationships.

#### Covariates

The following covariates from 9 months assessment were used in the analysis: sex, minority ethnicity, and self-reported highest level of education of either parent at participants’ birth. Ethnic minority group membership was measured with parent-reported ethnic minority status (0 = White British; 1 = Other). Parental education was defined by the highest educational level of either parent (0 = Obligatory education or lower; 1 = A level or vocational equivalent or higher education or university degree). Further, maternal mental health problems at 3 years of age were included as a covariate to assess whether maternal warmth predicts relationship problems over and above mental health [28]. Maternal mental health problems were assessed using the Kessler Psychological Distress Scale [29], which is a widely used brief screening tool for mental health problems in the general population. It includes 6 items (e.g., *‘How often did you feel hopeless?’*) rated on a 5-point scale ranging from ‘none’ to ‘all of the time’ that assess psychological distress in the past 30 days. Mean value was computed with high scores reflecting high symptoms of mental health problems.

### Statistical analysis

#### Missing data strategy

Sampling weights were applied to the analyses to account for the stratified clustered design of the data and the oversampling of subgroups. Missing data in all analyses was accounted for with multiple imputation using chained equations with the "mi" command in Stata. Each data set was imputed 20 times.

#### Main analyses

All statistical analyses were conducted with Stata, version 15.0 (StataCorp, 2017). Analysis of variance and χ^2^ tests were conducted to compare the VPT and MLPT groups with FT group on demographic variables, maternal mental health problems, maternal warmth, peer relationship problems and low engagement in romantic relationships. We conducted hierarchical linear regression analyses where we added all variables into the regression following a procedure recommended by Aiken and West (1991): 1) covariates and preterm birth (i.e., MLPT and VPT birth respectively); 2) maternal warmth; and 3) interaction terms (i.e., MLPT*maternal warmth, and VPT*maternal warmth respectively). The analyses were repeated for the MLPT and VPT groups separately and for the six outcome variables (i.e., self- and mother-reported peer relationship problems at 14 and 17 years, self-reported low engagement in romantic relationships at 14 and 17 years). For the regression analyses, the continuous maternal warmth variable was centered. All outcome variables were z-standardized. We applied the Benjamini–Hochberg procedure (i.e., using 25% false discovery rate) to correct for the influence of multiple comparisons [30].

## Results

Table 1 shows the characteristics of the sample according to the three birth groups (VPT, MLPT, FT). There were no differences between the three groups in maternal warmth at 3 years of age. However, PT born adolescents had higher peer relationship problems and lower romantic engagement than FT borns at all assessments except self-reported peer relationship problems at 14 years. Bivariate correlations between all study variables are shown in Supplementary Table 2.

**Table 1:** Participant characteristics by gestational age categories

	VPT (N = 99; 1.1%)	MLPT (N = 629; 6.8%)	FT (N = 8465; 92.1%)	p
Female: n(%)	49 (49.5%)	307 (48.8%)	4383 (51.8%)	.33
Minority: n(%)	28 (28.3%)	116 (18.4%)	1503 (17.8%)	.02
Parental education below tertiary: n(%)	17 (17.2%)	147 (23.4%)	1701 (20.1%)	.11
Maternal mental health problems at 3 years	.73 (.08)	.73 (.03)	.67 (.01)	.29
Maternal Warmth at 3 years	26.30 (2.26)	26.40 (2.46)	26.55 (2.41)	.29
Self-reported peer relationship problems at 14 years	1.74 (1.90)	1.86 (1.89)	1.77 (1.84)	.55
Mother-reported peer problems at 14 years	2.25 (1.97)	1.83 (1.90)	1.67(1.79)	.002
Self-reported peer problems at 17 years	2.49 (1.71)	2.30 (1.82)	2.12 (1.70)	.01
Mother-reported peer problems at 17 years	2.46 (2.21)	1.86 (1.89)	1.69 (1.77)	< .001
Self-reported low engagement in romantic relationships at 14 years	6.85 (1.27)	6.52 (1.42)	6.39 (1.42)	.002
Self-reported low engagement in romantic relationships at 17 years	3.60 (.71)	3.38 (.80)	3.27 (.82)	< .001

*VPT* very preterm, *MLPT* moderate to late preterm, *FT* full-term

### Associations between moderate to late preterm birth, maternal warmth and peer relationship problems and engagement in romantic relationships

**Fig. 1:**
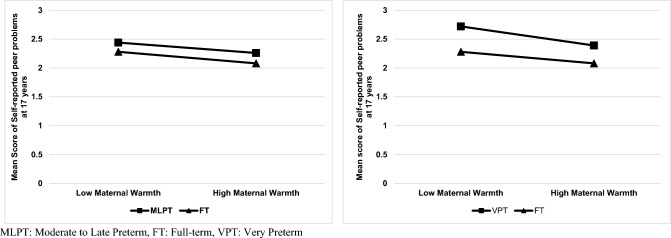
Interaction between preterm birth and maternal warmth in early childhood on self-reported peer relationship problems at 17 years of age

MLPT birth was associated with lower engagement in romantic relationships at 17 years of age than FT birth (b = 0.04, p = 0.02) (Table 2; See Supplementary Table 3 for full findings). Higher levels of maternal warmth at 3 years of age were associated with lower mother- (b = -0.19, p < 0.001) and self-reported peer relationship problems at 14 years (b = -0.10, p = 0.01), as well as mother- (b = -0.09, p < 0.001) and self-reported peer relationship problems at 17 years (b = -0.13, p < 0.001). Moreover, higher levels of maternal warmth at 3 years of age were associated with lower problems with engagement in romantic relationships at 14 years of age (b = -0.04, p = 0.02). Maternal warmth at 3 years moderated the association of MLPT birth with self-reported peer relationship problems at 17 years of age (b = 0.10, p = 0.03) (Fig. 1). However, the slopes for MLPT and FT groups were similar, and the moderation effect was not significant after adjustment for multiple comparisons and when using multiple imputed data (See Supplementary Table 5). There was no other significant moderation effect.

**Table 2:** Associations between moderate to late preterm birth, maternal warmth, peer relationship problems and low romantic engagement in adolescence

		Peer relationship problems								Low Romantic Engagement			
		Mother report at 14 years		Self-report at 14 years		Mother report at 17 years		Self-report at 17 years		Self-report at 14 years		Self-report at 17 years	
Steps		b	p	b	p	b	p	b	p	**b**	**p**	**b**	**p**
1	MLPT birth	.08	.06	.01	.74	.01	.74	.06	.19	.04	.29	**.04**	**.02**
2	MLPT birth	.06	.13	.02	.71	.01	.86	.04	.32	.03	.37	.04	.03
	Maternal Warmth at 3 years	−**.19**	** < .001**	−**.10**	**.01**	−**.09**	** < .001**	−**.13**	** < .001**	−**.04**	**.02**	−.002	.83
3	MLPT birth	.06	.13	.02	.66	.01	.89	.04	.34	.03	.36	.04	.03
	Maternal Warmth at 3 years	−.19	< .001	−.01	.94	−.04	.37	−.04	.34	-.003	.92	-.004	.84
	MLPT x Maternal Warmth at 3 years	.01	.86	.11	.14	.05	.25	**.10**	**.03**	.04	.23	-.002	.92

MLPT: 1 = Moderate to late preterm, 0 = Full-term; b = regression coefficient (standardized)

Boldface indicates a significant association. N = 8564

Please note that the main effect of MLPT birth was based on the results from step 1, and the main effect of maternal warmth was reported using step 2 findings

### Associations between very preterm birth, maternal warmth and peer and romantic relationship problems

VPT birth was associated with mother-reported peer relationship problems at 14 years (b = 0.29, p = 0.01), and low engagement in romantic relationships at 17 years (b = 0.11, p = 0.02). The association between VPT birth and self-reported peer relationship problems at 17 years (b = -0.22, p = 0.046) was not significant after correcting for multiple comparisons (Table 3; See Supplementary Table 4 for full findings). Higher levels of maternal warmth at 3 years of age were associated with lower mother- (b = -0.19, p = 0.01) and self-reported peer relationship problems at 14 years (b = -0.06, p = 0.009), as well as mother- (b = -0.09, p < 0.001) and self-reported peer relationship problems at 17 years (b = -0.14, p < 0.001). Moreover, higher levels of maternal warmth at 3 years of age were associated with lower engagement in romantic relationships at 14 years of age (b = -0.05, p = 0.01). It was shown that maternal warmth at 3 years moderated the association of VPT birth with self-reported peer relationship problems at 17 years of age (b = 0.29, p = 0.03). Nevertheless, the slopes for VPT and FT groups were similar, and the moderation effect was not statistically significant after correction for multiple comparisons (Fig. 1). There was no other significant moderation effect. The main findings about the moderation effect did not change using multiple imputed data (See Supplementary Table 6).

**Table 3:** Associations between very preterm birth, maternal warmth, peer relationship problems and low romantic engagement in adolescence

Steps		Peer Relationship Problems						Low Romantic Engagement					
		Mother report at 14 years		Self-report at 14 years		Mother report at 17 years		Self-report at 17 years		Self-report at 14 years		Self-report at 17 years	
		b	p	b	p	b	p	b	p	b	p	b	p
1	VPT birth	**.29**	**.01**	.07	.55	.08	.47	**.22**	**.046**	.12	.15	**.11**	**.02**
2	VPT birth	.27	.01	.04	.72	.09	.39	.21	.06	.14	.11	.11	.02
	Maternal Warmth at 3 years	−**.19**	**.01**	−**.06**	**.009**	−**.09**	** < .001**	−**.14**	** < .001**	−**.05**	**.01**	−.001	.90
3	VPT birth	**.29**	**.01**	.04	.71	.12	.28	.22	.06	.14	.10	**.11**	**.03**
	Maternal Warmth at 3 years	.02	.86	−.06	.64	.08	.45	.15	.26	.04	.68	.03	.62
	VPT x Maternal Warmth at 3 years	.22	.05	.00	.99	.17	.10	**.29**	**.03**	.09	.39	.03	.60

*VPT* very preterm, b = regression coefficient (standardized)

Boldface indicates a significant association. N = 9094

Please note that the main effect of VPT birth was based on results from step 1, and the main effect of maternal warmth was reported using step 2 findings

## Discussion

Findings from the current study showed that VPT birth as opposed to FT birth was associated with higher peer relationship problems in adolescence. Maternal warmth in early childhood had a protective influence on peer relationship problems similarly in both PT and FT adolescents. Furthermore, both MLPT and VPT birth were associated with lower engagement in romantic relationships in late adolescence. However, maternal warmth in early childhood did not alter the magnitude of low engagement in romantic relationships. Moreover, maternal warmth in early childhood did not change the magnitude of the association between PT birth and problems in peer relationships and low engagement in romantic relationships.

In line with the findings of previous studies, the current study revealed that adolescents born VPT are more likely to experience peer relationship problems [11, 12, 14]. Our findings suggest that there is no major modifying effect of early maternal warmth for the association between VPT birth and peer relationship problems in late adolescence. No significant interaction effect was found after adjustment for multiple comparisons and inspection of the figure and slopes for VPT and FT adolescents is consistent with the finding that maternal warmth has a similar protective influence for VPT and FT adolescents. This finding is in contrast to the findings of the previous studies which showed that positive parenting can modify the association between VPT birth and problems in socio-emotional competence in early childhood [18, 31] and other outcomes such as problems in academic achievement at 13 years of age [22]. The modifying effect of maternal warmth on the outcomes (particularly peer relationship problems) of VPT birth may not be evident in early childhood.

Rather, maternal warmth in early childhood is beneficial for both PT and FT born adolescents similarly in lowering peer relationship problems even when the role of maternal mental health problems is considered. This finding could be explained by two theories. The first explanation could be made via social learning theory [32], which suggests that children transfer behavioral and relationship patterns that they learn in their family interactions to other social conditions such as peer relationships [33]. Indeed, a meta-analytic investigation showed that positive parenting (i.e., authoritative parenting, communication, involvement, warm and affectionate relationship, and support) during childhood was associated with fewer peer relationship problems (i.e., bullying and victimization) in FT samples [34]. Another explanation for the protective role of maternal warmth could be made via attachment theory suggesting that the quality of the early parent–child attachment relationship influences the development of positive internal working models of the self and others and emotion regulation, which influence the way children approach future social situations [35]. Thus, maternal warmth in early childhood could increase both PT and FT adolescents’ capacity to cope with negative affect, thereby altering their experience and/or expectations of social interactions [36, 37]. This was supported by a meta-analysis study which showed that parent–child attachment security was associated with higher levels of competence in peer relationships [38].

Our findings showed that both MLPT and VPT born children were less likely to engage in romantic relationships during late adolescence. This association was weak for MLPT adolescents and slightly stronger for VPT adolescents. This could be explained by the behavioural and personality phenotype associated with PT birth which includes being socially withdrawn and disinclined towards risk-taking or fun seeking behaviors, [39] which could predispose PT born adolescents to have less contact with their peers, fewer friends [40], and less likely to meet potential partners for romantic relationships than FT born adolescents. Maternal warmth in early childhood had a protective influence on low engagement in romantic relationships in early adolescence but not in late adolescence. This suggests that, for PT adolescents, maternal warmth in early childhood may be less important for romantic relationship engagement in late adolescence during which the romantic relationships may become more exclusive including more steady involvement with one partner in comparison to early adolescence which may include group dates [41]. This finding is in line with the suggestion that it might take longer for PT born individuals to form romantic relationships [42]. It was found in a meta-analysis that PT adults aged from 18 to 25 years are 50% less likely than FT borns to ever experience sexual intercourse [16]. Our findings provide further support to the evidence that PT birth is associated with lower likelihood in romantic engagement, which is already evident in late adolescence although the strength of the associations is weak.

There are several strengths of the current longitudinal study including the large sample size representative of the population in the UK and using both self- and mother-reports of peer relationship problems. On the other hand, there are limitations of the current study. First, our measure of romantic relationships in adolescence did not include the quality of the romantic relationships and only reflects engagement in romantic relationships. Second, the measurements of peer relationship problems and engagement in romantic relationships differed at 14 and 17 years, which potentially influenced the findings. However, the 14-year measurements were correlated with the measurements at 17 years, suggesting that the assessment was reliable. Third, maternal warmth in early childhood was reported by mothers which might have been influenced by social desirability. It was shown that the maternal warmth sub-scale used in this study was significantly associated with observed mother–child relations, however the association was weak [43]. Observations of parent–child interactions are considered as the ‘gold standard’ for assessing parenting behaviors, however conducting such measurements require considerable time and financial resources, which makes it difficult to apply in population based prospective studies [44]. Fourth, we cannot rule out the role of unmeasured confounding variables on our findings such as the role of religion.

## Conclusion

To conclude, adolescents born VPT have more peer relationship difficulties than their peers born at term. Maternal warmth in early childhood can protect both PT (both MLPT and VPT) and FT children from these difficulties. Furthermore, the association between PT birth (both MLPT and VPT) and lower engagement in romantic relationships is already evident in late adolescence. Maternal warmth has a protective influence on engagement in romantic relationships at 14 years of age in both MLPT and VPT adolescents, but not at 17 years of age. Future research is required to investigate the role of other potential factors that may promote social outcomes in PT born adolescents such as personality traits, engagement in leisure activities, and secure attachment.

## Abbreviations

PT: Preterm
MLPT: Moderate-to-late preterm
VPT: Very preterm
FT: Full-term
MCS: Millennium Cohort Study
